# Amino acid substitutions in a polygalacturonase inhibiting protein (OsPGIP2) increases sheath blight resistance in rice

**DOI:** 10.1186/s12284-019-0318-6

**Published:** 2019-07-29

**Authors:** Xijun Chen, Yuwen Chen, Lina Zhang, Zhen He, Benli Huang, Chen Chen, Qingxia Zhang, Shimin Zuo

**Affiliations:** 1 Horticulture and Plant Protection College, Yangzhou University, Yangzhou, 225009 China; 2 Key Laboratory of Crop Genetics and Physiology of Jiangsu Province/Key Laboratory of Plant Functional Genomics of the Ministry of Education, College of Agriculture, Yangzhou University, Yangzhou, 225009 China

**Keywords:** Polygalacturonase inhibiting proteins, Amino acid substitution, Sheath blight resistance, Rice

## Abstract

**Background:**

An economic strategy to control plant disease is to improve plant defense to pathogens by deploying resistance genes. Plant polygalacturonase inhibiting proteins (PGIPs) have a vital role in plant defense against phytopathogenic fungi by inhibiting fungal polygalacturonase (PG) activity. We previously reported that rice PGIP1 (OsPGIP1) inhibits PG activity in *Rhizoctonia solani*, the causal agent of rice sheath blight (SB), and is involved in regulating resistance to SB.

**Result:**

Here, we report that OsPGIP2, the protein ortholog of OsPGIP1, does not possess PGIP activity; however, a few amino acid substitutions in a derivative of OsPGIP2, of which we provide support for L233F being the causative mutation, appear to impart OsPGIP2 with PG inhibition capability. Furthermore, the overexpression of mutated *OsPGIP2*^*L233F*^ in rice significantly increased the resistance of transgenic lines and decreased SB disease rating scores. *OsPGIP2*^*L233F*^ transgenic lines displayed an increased ability to reduce the tissue degradation caused by *R. solani* PGs as compared to control plants. Rice plants overexpressing *OsPGIP2*^*L233F*^ showed no difference in agronomic traits and grain yield as compared to controls, thus demonstrating its potential use in rice breeding programs.

**Conclusions:**

In summary, our results provide a new target gene for breeding SB resistance through genome-editing or natural allele mining.

**Electronic supplementary material:**

The online version of this article (10.1186/s12284-019-0318-6) contains supplementary material, which is available to authorized users.

## Background

Rice is the most widely-consumed staple food worldwide and feeds over half the world population, particularly in Asia (Kumar et al. 2009; Li et al. 2017). Sheath blight (SB) caused by *Rhizoctonia solani*, is one of the most serious diseases of rice and recently caused large production losses in the Yangtze River region of China (Wang et al. 2015; Dey et al. 2016). *R. solani* is a broad-host range saprophyte, and there are no rice cultivars with complete immunity to SB (Eizenga et al. 2002; Hossain et al. 2014; Zhu et al. 2014). Partial resistance exists in some rice cultivars and is conferred by polygenes encoded by quantitative trait loci (QTLs). Introgression of SB resistance via QTLs or by introducing SB-related defense genes has resulted in improved SB resistance in rice (Gaihre et al. 2015; Richa et al. 2016). However, only a few genes or QTLs with high breeding potential have been identified for SB (Zuo et al. 2008, 2011; Zhu et al. 2014).

For successful colonization of plant tissues, pathogens must breach plant cell wall, the first barrier of defense against pathogens. Nearly all fungi, particularly necrotrophic pathogens, must initially secrete PGs to dissolve pectin and related components of plant cell wall (Jones and Jones 1997; Isshiki et al. 2001; Protsenko et al. 2008; Zhang et al. 2014). For example, mutants of *Claviceps purpurea* lacking the PGs encoded by *cppg1* and *cppg2* were almost completely nonpathogenic on rye (Oeser et al. 2002).

As a counteroffensive strategy, plants have evolved the ability to secrete PGIPs to reduce the degradation activity of PGs on pectin and related compounds. Complexes between PGIPs and PGs are considered a model system of enzyme-inhibitor interactions at the plant/pathogen interface (Masas-Villamil and Van der Hoorn 2008). It has been previously demonstrated that single amino acid substitutions in PGs may enable the pathogen to escape PGIP recognition; similarly, amino acids substitutions in PGIPs may increase inhibitory properties with respect to PGs (Bishop 2005; Benedetti et al. 2013). In addition to their function in PG inhibition, PGIPs promote the accumulation of oligogalacturonide (OGs) elicitors, which induce host defense responses (Federici et al. 2006; Ferrari et al. 2013). Transgenic plants partially silenced for *PGIP* genes expression showed enhanced susceptibility to fungal infection, whereas transgenic plants overexpressing *PGIP* genes displayed more resistance to fungi (Ferrari et al. 2006; Chen et al. 2016; Liu et al. 2016). These studies demonstrate the important roles of PGIPs in the modulation of plant defense to fungal ingress.

Numerous *PGIP* genes have been identified based on the conserved domain “xxLxLxxNxLxGxIPxxLxxLxxL”; however, only a few have been well investigated (Manfredini et al. 2005; D’Ovidio et al. 2006; Kalunke et al. 2015; Kumar 2017). Some *PGIP* genes share high sequence identity, whereas others do not (Kalunke et al. 2015). Furthermore, different PGIPs, including members in the same gene subfamily, exhibit diverse inhibitory activities on PGs from same or different pathogens (Desiderio et al. 1997; Stotz et al. 2000; Sicilia et al. 2005). Bean (*Phaseolus vulgaris*) harbors four PGIPs (PvPGIP1–4) that differ in efficacy against PGs produced by plant pathogens. PvPGIP1 and PvPGIP2 have only eight different amino acids; however, PvPGIP2 has strong PG-inhibiting activity but PvPGIP1 does not (Bishop 2005). In rice, seven *OsPGIP* genes are reported; four (*OsPGIP1*-*OsPGIP4*) are mapped to a 30-kb region on chromosome 5 and the remaining three are mapped to chromosomes 7, 8 and 9 (Janni et al. 2006). *OsPGIP* genes show various expression patterns in different tissues and organs and are differentially expressed in response to phytohormones or stresses (Janni et al. 2006; Lu et al. 2012; Chen et al. 2016). The expression level of *OsPGIP1* in resistant cultivar YSBR1 is highly elevated under different abiotic and biotic stresses (Chen et al. 2016). OsPGIP1 is shown to inhibit PG from *Rhizoctonia solani* (RsPG) activity (Jang et al. 2003; Janni et al. 2006; Wang et al. 2015), and the overexpression of *OsPGIP1* significantly improves rice resistance to SB (Wang et al. 2015; Chen et al. 2016). The OsPGIP homologue OsFOR1 inhibits the activity of PGs from *Aspergillus niger* (Jang et al. 2003); however, there was no evidence indicating a role in plant defense to fungal pathogens. In general, the roles of OsPGIPs in rice defense against pathogens remains to be elucidated.

In the present study, we showed that OsPGIP2 harbored ten tandem repeats of the canonical 24 amino acid LRR sequence, and each repeat contained the consensus “xxLxLxx” motif; however, unlike OsPGIP1, OsPGIP2 did not inhibit RsPGs. We used site-directed mutagenesis to develop amino acid substitutions in OsPGIP2; we identified one mutant designated OsPGIP2^L233F^ that inhibited RsPGs. *OsPGIP2*^*L233F*^ was overexpressed in rice cultivar Xudao No. 3 (cv. XD3) and transgenic rice lines displayed higher SB resistance as compared with the control. Our results indicate that *OsPGIP2* is an ideal target for increasing SB resistance in rice.

## Results

### Characterization of OsPGIP2

OsPGIP2 contains 342 amino acids with ten tandem leucine rich repeat (LRR) units; the deduced protein shows typical PGIP topology with a putative signal peptide of 22 amino acids. The molecular weight and pI of deduced OsPGIP2 were 37.0 kD and 4.73, respectively, and the primary structure was hydrophobic (hydrophobic coefficient of 0.102). OsPGIP2 contained a leucine-rich nuclear export signal sequence (LLLLLSVLLL) and transmembrane helix, which suggests an extracellular location. This was congruent with the predicted localization of the protein, which suggested that OsPGIP2 was secreted. The instability index of OsPGIP2 was 40.41, giving a hint that the protein is stable. The aliphatic index and grand average of hydropathicity suggested that OsPGIP2 is hydrophobic. The predicted solubility of OsPGIP2 overexpressed in *E. coli* was 0.0%, implying that overexpression products will likely form inclusion bodies (Table 1).

**Table 1:** Characterization of deduced OsPGIP2 protein

Characteristic		Predicted Results	Characteristic	Predicted Results
Gene ontology		Leucine-rich repeat (LRR) protein	Transmembrane helices	Extracellular
Number of amino acids		342	Subcellular location	Secretory pathway
Molecular weight		36966.91	Instability index	40.41
Theoretical pI		4.73	Aliphatic index	103.33
Cleavage site of signal peptide		Between 22th and 23th amino acid	Grand average of hydropathicity	0.08
Secondary structure	Alpha helix	47.66%	Estimated half-life	30 h (mammalian reticulocytes, in vitro)
	Extended strand	10.23%		> 20 h (yeast, in vivo)
	Random coil	42.11%		> 10 h (*E. coli*, in vivo)
Leucine-rich nuclear export signals		LLLLLSVLLL	Solubility when overexpressed in *E. coli*	0.0%

**Fig. 1:**
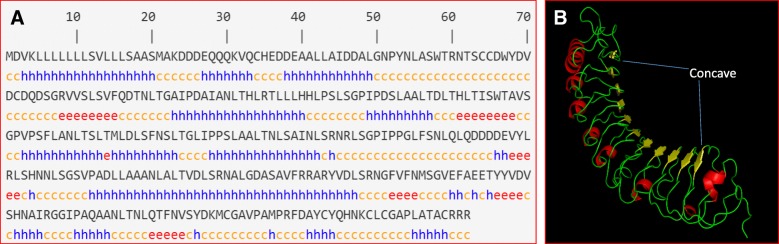
The secondary and tertiary structure of deduced OsPGIP2. **a**, Secondary structure of OsPGIP2. Lowercase c, h and e represent random coils, α-helices and extended strands, respectively. **b**, Tertiary structure of OsPGIP2 predicted by homology modeling. α-helices are shown in red, and β-strands are indicated in yellow.

Secondary structure prediction showed that OsPGIP2 contains *ɑ*-helixes, *β*-sheets and irregular coils, but no coiled-coil motifs (Table 1 and Fig. 1a). A 3D-model of the tertiary structure predicted by homology modeling indicated that OsPGIP2 has highest identity with *Phaseolus vulgaris* PGIP2 (PvPGIP2) and could form a cleft that functions to inactivate PGs produced by pathogens (Fig. 1b).

### Selection of sites for mutagenesis

**Fig. 2:**
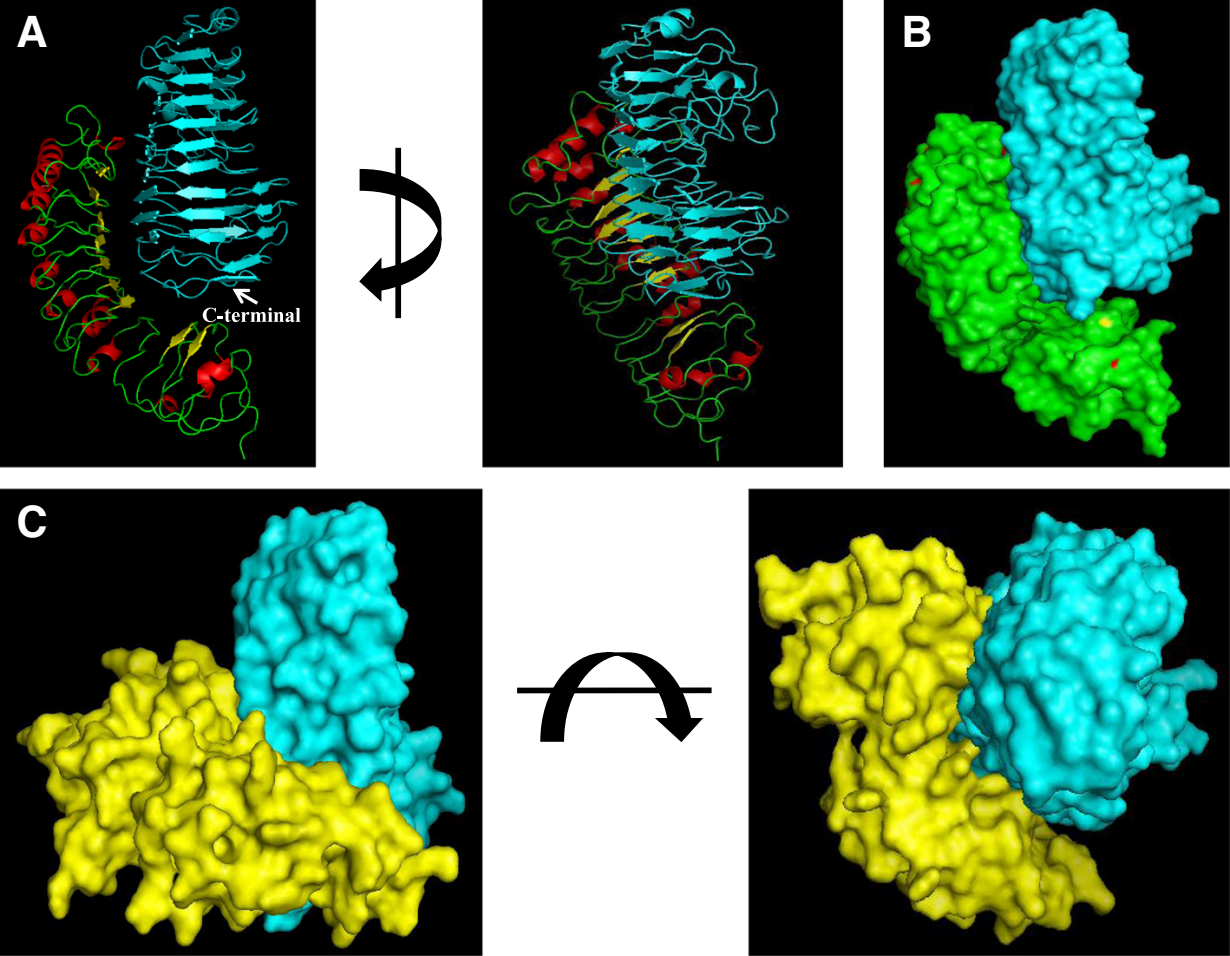
Comparative analysis of OsPGIP1-RsPG1 and OsPGIP2-RsPG1. **a**, Cartoon representation of the complex formed by OsPGIP1 (red and yellow) and RsPG1 (cyan blue). Two orthogonal perspectives are shown. **b**, Surface representation of the complex formed by OsPGIP1 (green) and RsPG1 (cyan). **c**, Surface representation of the complex formed by OsPGIP2 (yellow) and RsPG1 (cyan). Two orthogonal views are shown.

Previous studies showed that single amino acid substitutions in PGIPs or PGs could inhibit or change their inherent properties (Leckie et al. 1999; Benedetti et al. 2013). Docking analysis of the interaction between OsPGIP1 or OsPGIP2 and RsPG1 showed that the binding sites of OsPGIP1 or OsPGIP2 are located in the cleft, and that RsPG1 is engaged at the C-terminal edge (Fig. 2). Although preliminary experiments indicated that OsPGIP2 could not inhibit RsPG activity, modeling indicated that OsPGIP2 could form a complex with RsPG1 (Fig. 2).

**Fig. 3:**
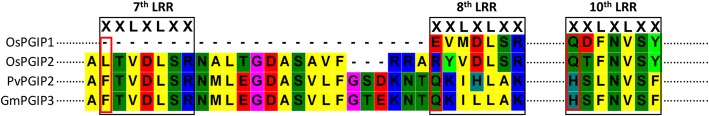
Alignment of OsPGIP1, OsPGIP2, PvPGIP2 and GmPGIP3 amino acid sequences. LRR motifs associated with extracellular or extracytoplasmic location are indicated by the consensus XXLXLXX motifs. The amino acids in OsPGIP2 that differ from the corresponding residues in OsPGIP1, PvPGIP2 and GmPGIP3 are bordered with red rectangles. Dashed (−) lines represent gaps in OsPGIP1 and OsPGIP2; dotted lines represent the omitted amino acids in all four PGIPs

OsPGIP1, PvPGIP2 and GmPGIP3 (PGIP3 from *Glycine max*) exhibited strong inhibition for PGs from the corresponding pathogens, such as *Sclerotinia sclerotiorum*, *Fusarium graminearum*, *Botrytis cinerea* and *Aspergillus niger* (Janni et al. 2006; Maulik et al. 2009). We compared the deduced amino acid sequences of OsPGIP1 and OsPGIP2 and observed that the seventh LRR motif of OsPGIP2 was absent in OsPGIP1 (Janni et al. 2006). Furthermore, OsPGIP2 contained one amino acid substitution (L233) that is a phenylalanine residue in PvPGIP2 and GmPGIP3, which both have strong PGIP activity. The Q253 residue in PvPGIP2 had a major contributory effect on inhibiting the activity of PGs from *Fusarium moniliforme*, and its replacement with lysine resulted in a dramatic decrease in PGIP activity (Leckie et al. 1999). Conversely, PvPGIP1 acquired the ability to interact with *F. moniliforme* PGs when the 253rd amino acid residue was mutated from lysine to glutamine; however, the 253rd amino acid in OsPGIP2 was arginine. Furthermore, histidine is present in the active center of many functional PGIPs at the 10th LRR (e.g. PvPGIP2 and GmPGIP3), but OsPGIP2 contains a glutamine at this location (Fig. 3). Based on these findings, L233, R253, and Q300 in OsPGIP2 were selected as the targets to change into phenylalanine, glutamine and histidine, respectively.

### PGIP activity of wild-type and mutated forms OsPGIP2

**Fig. 4:**
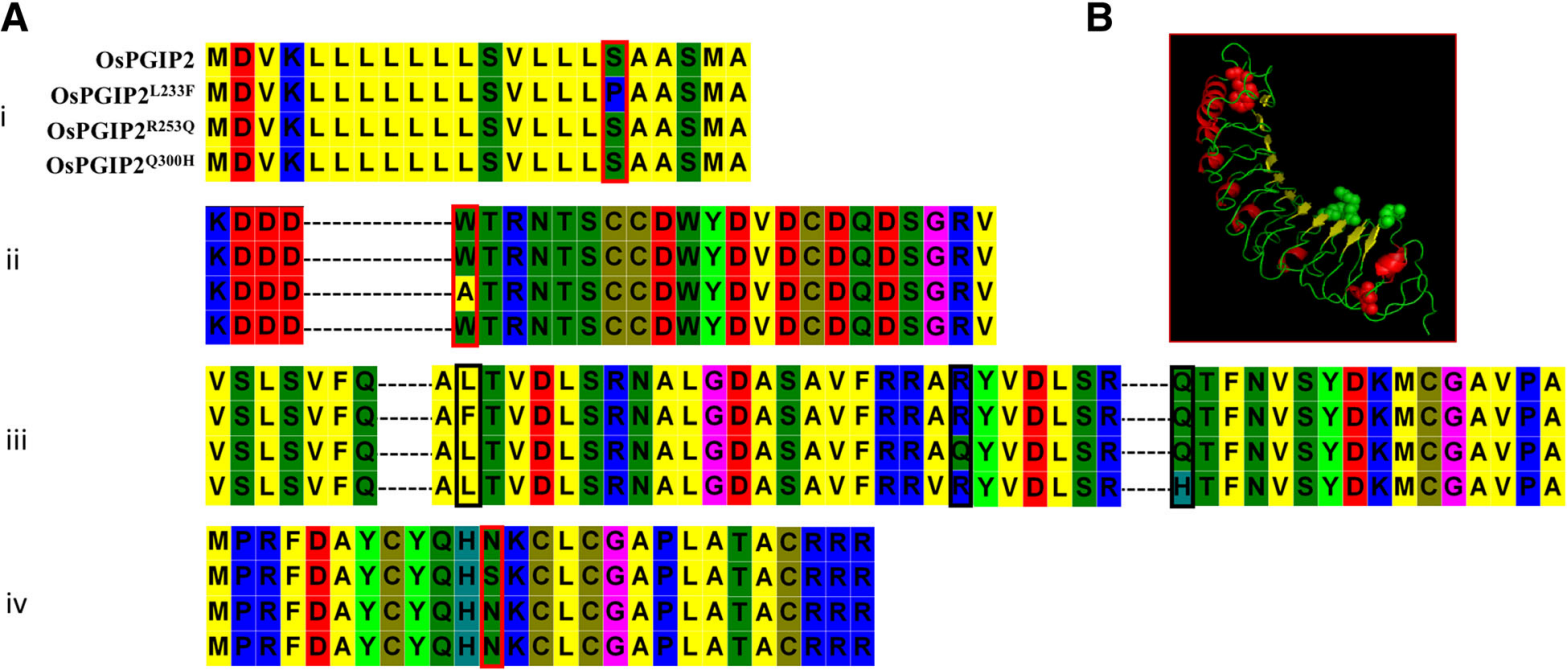
Substituted and mismatched amino acids in wild-type OsPGIP2 and mutated variants OsPGIP2^L233F^, OsPGIP2^R253Q^, and OsPGIP^Q300H^. **a**, Amino acid sequence of OsPGIP2, OsPGIP2^L233F^, OsPGIP2^R253Q^, and OsPGIP^Q300H^. (i) Signal peptide; (ii), predicted N-terminus of mature proteins; (iii) LRR motifs; and (iv), C-terminus. Black and red rectangles represent targeted and unanticipated mutations, respectively. **b**, Cartoon model of OsPGIP2. Red and green balls denote mismatched and substituted amino acids, respectively.

Wild-type *OsPGIP2* and the three mutant forms, *OsPGIP2*^*L233F*^, *OsPGIP2*^*R253Q*^, and *OsPGIP*^*Q300H*^, were amplified from cDNA of rice cv. XD3, cloned in pET22b, and sequenced. Sequence analysis confirmed the mutation of L233, R253, and Q300 in OsPGIP2^L233F^, OsPGIP2^R253Q^, and OsPGIP^Q300H^ to phenylalanine, glutamine, and histidine, respectively (Fig. 4). We also found two other amino acid substitutions (S17P and N327S) in OsPGIP2^L233F^ and one more substitution (W57A) in OsPGIP2R253Q but they all located in non-activity region of OsPGIP (Fig. 4).

**Fig. 5:**
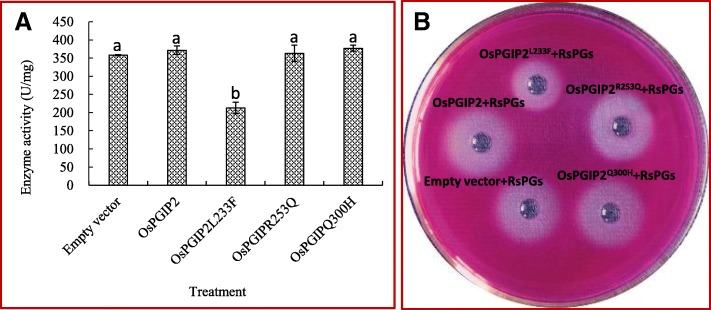
Reduced sugar and diffusion assays for inhibition of RsPG activity. OsPGIP2 and mutant forms (OsPGIP2^L233F^, OsPGIP2^R253Q^, and OsPGIP2^Q300H^) were overproduced in *E. coli* BL21 and assayed for inhibition of RsPGs. **a**, The reduced sugar assay; **b**, The agar diffusion assay. Different lowercase letters on the bar show significant statistical difference between treatment and control at 5% significance level. Each bar represents the average and standard deviation of at least three samples.

Recombinant OsPGIP2, OsPGIP2^L233F^, OsPGIP2^R253Q^, and OsPGIP^Q300H^ were extracted, purified, and used to evaluate inhibition of RsPGs (Chen et al. 2016). OsPGIP2 and mutant derivatives were insoluble when overproduced in *E. coli* BL21; however, after disrupting the bacterial cells by sonication and refolding the proteins, they were used in the reduced sugar and agar diffusion assays (Fig. 5). Except for OsPGIP2^L233F^ protein, which resulted in a reduction of 40.65% of RsPG activity, OsPGIP2, OsPGIP2^R253Q^ and OsPGIP^Q300H^ showed no significant inhibiting activity on RsPGs. In the agar diffusion assay, it was clearly evident that the diameter of the diffusion ring surrounding OsPGIP2^L233F^ plus RsPGs was smaller than the other recombinant protein/RsPG mixtures (Fig. 5b).

### Overexpression of *OsPGIP2*^*L233F*^ in rice increases SB resistance

**Fig. 6:**
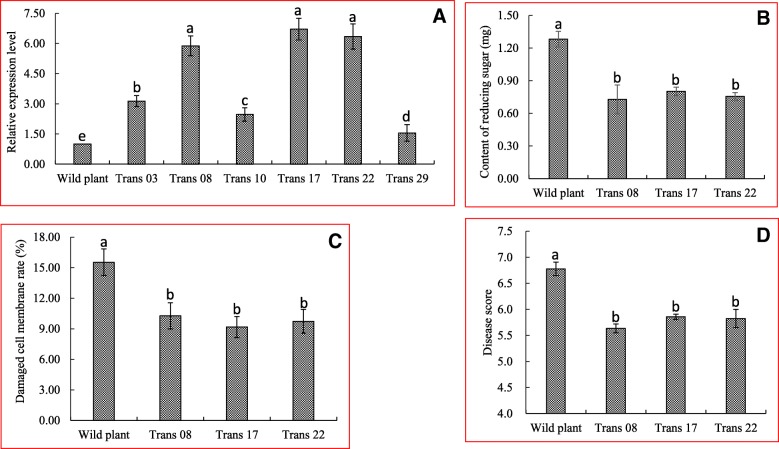
Analysis of transgenic lines expressing *OsPGIP2*^*L233F*^. **a**, Relative expression level of *OsPGIP2* in wild-type cv. XD 3 and the six transgenic lines (Trans 03, 08, 10, 17, 22, and 29) containing *OsPGIP2*^*L233F*^. **b**, Content of reducing sugars in wild-type and transgenic rice lines (Trans 08, 17, and 22); seedlings were immersed in a solution containing RsPGs. **c**, Cell membrane damage (%) as measured by electrical conductivity in wild-type and transgenic plants (%). **d**, Disease rating scores in wild-type and transgenic lines inoculated with *R. solani* YN-7. Different lowercase letters on the column show significant statistical difference between treatment and control at 5% significance level. Each bar represents the average and standard deviation of at least three samples.

PGIP activity assays suggested that OsPGIP2^L233F^ might be utilized in transgenic rice to increase resistance to tissue degradation by RsPGs, thereby improving resistance to *R. solani*. To test this hypothesis, we overexpressed the mutant gene *OsPGIP2*^*L233F*^ in cv. XD3, which is susceptible to *R. solani* and widely planted in the Jiangsu province of China. Thirty independent transgenic plants were obtained by *Agrobacterium*-mediated transformation, and the introduction of *OsPGIP2*^*L233F*^ was indirectly confirmed by PCR using primers specific to the *HPT* gene (Additional file 1: Figure S1). Six lines with a single copy of the transgene (Trans 03, 08, 10, 17, 22, and 29) were identified by analyzing the segregation of T_1_ plants relative to T_0_ lines; a 3:1 ratio was considered evidence for a single copy of the introduced transgene (Additional file 2: Table S1). Three lines, Trans 08, 17, and 22, retained the typical agronomic traits of wild-type plants and were developed into homozygous lines. The homozygous lines showing strong expression of *OsPGP2*^*L233F*^ were selected for further experiments (Fig. 6a).

PGs, from the pathogenic agents, generally function in resolving polysaccharide into reducing sugar, resulting in the damage of cell walls and membranes of plants. For testing the ability of transgenic plants against RsPGs, we immersed the rice sheath into the RsPGs solution, and found that the reducing sugar content from three transgenic lines were 0.73 ± 0.02, 0.75 ± 0.02 and 0.80 ± 0.08 mg/mL, respectively, and all significantly lower than that from wild-type plants (1.28 ± 0.04 mg/mL) (Fig. 6b). We also found that the percentage of damaged cell membranes of transgenic lines (ranged from 9.17 ± 0.59 to 10.27 ± 0.74) were significantly lower than that of wild type (15.53 ± 0.75) (Fig. 6c). When homozygous transgenic lines and the wild-type XD 3 were inoculated with *R. solani* YN-7 at late tillering in field, the *OsPGIP2*^*L233F*^ overexpression lines showed significantly lower disease scores (ranged from 5.63 ± 0.05 to 5.86 ± 0.03) than the control (6.78 ± 0.07), reducing the disease score by 0.92 to 1.14 (Fig. 6d). Collectively, these results indicate that OsPGIP2^L233F^ expressed in transgenic plants, inhibited RsPGs, and enhanced rice resistance to sheath blight.

### *OsPGIP2*^*L233F*^ has no deleterious effects on rice growth and development

**Fig. 7:**
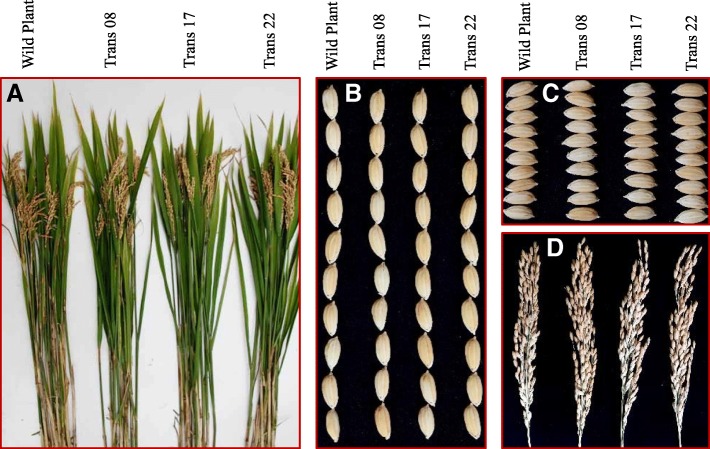
Phenotype of wild-type cv. XD3 and transgenic lines, Trans 08, 17, and 22. **a**, Plants inoculated with *R. solani* YN-17. Panels **b**, **c**, and **d** show seed length, width, and panicles.

To determine the potential of *OsPGIP2*^*L233F*^ for implementation in rice breeding programs, we measured a set of agronomic traits in the three transgenic homozygous lines, Trans 08, 17 and 22. Results showed that there were no significant differences among the transgenic lines and the wild-type cv. XD 3 in agronomic traits, including heading date, plant height, flag leaf length and width, and tiller numbers. Furthermore, no differences were observed between the wild-type and the transgenic lines with respect to yield-associated components, including the number of productive panicles, panicle length, the number of the primary branches, seed number/panicle, grain length and width, and 1,000-grain weight (Fig. 7**,** Table 2 and Table 3).

**Table 2:** Agronomic characteristics of transgenic homozygous lines population

Treatment*	Plant height	Tiller number	Flag leaf length	Flag leaf width	Panicle length	Number of primary branches
Wild-type Xudao 3	96.46 ± 0.60	9.03 ± 0.29	21.12 ± 0.33	1.77 ± 0.03	19.35 ± 0.40	13.43 ± 0.19
Trans 08	96.15 ± 0.20	9.16 ± 0.27	20.65 ± 0.53	1.81 ± 0.03	19.20 ± 0.08	12.97 ± 0.22
Trans 17	95.55 ± 0.79	9.03 ± 0.18	21.03 ± 0.15	1.83 ± 0.03	18.93 ± 0.24	13.07 ± 0.07
Trans 22	95.88 ± 0.79	9.33 ± 0.20	21.16 ± 0.14	1.82 ± 0.02	19.11 ± 0.13	13.03 ± 0.19
*F* and *P* values	*F* = 0.1645 *P* = 0.2693	*F* = 0.4230 *P* = 0.7368	*F* = 1.4320 *P* = 0.3101	*F* = 0.0960 *P* = 0.9094	*F* = 0.9880 *P* = 0.4256	*F* = 0.5070 *P* = 0.6261

*F and *P* values were derived from analysis of variance. *P* values > 0.05 indicate no statistically significant difference. Numerical values in the table represent mean ± standard deviation

**Table 3:** Values of yield-associated components collected from populations of the homozygous transgenic lines

Treatment	Panicle number per plant	Seed number per panicle	1,000 seed-grain weight
Wild-type	8.54 ± 0.20	129.94 ± 2.73	27.29 ± 0.60
Trans 08	8.67 ± 0.39	131.36 ± 1.71	27.08 ± 0.54
Trans 17	8.62 ± 0.13	130.06 ± 1.69	26.78 ± 0.39
Trans 22	8.33 ± 0.31	131.31 ± 1.02	27.23 ± 0.08
*F* and *P*	*F* = 0.8430, *P* = 0.5077	*F* = 0.5000, *P* = 0.6925	*F* = 0.7590, *P* = 0.5477

*F* and *P* values were derived from analysis of variance. *P* values > 0.05 indicate no statistically significant difference. Numerical values in the table represent mean ± standard deviation

## Discussion

The PGIP-PG interaction is a classic model system for analyzing the complex evolution of protein inhibition and enzyme recognition in plant-pathogen interactions. In many plant species, such as rice (Janni et al. 2006), wheat (Janni et al. 2006), bean (D’Ovidio et al. 2004), soybean (Kalunke et al. 2014), rapeseed (Hegedus et al. 2008), pepper (Wang et al. 2013) and Arabidopsis (Ferrari et al. 2003), PGIPs exist as a protein family. Pathogens have evolved different PGs to maximize their offensive potential; conversely plants have evolved various PGIPs with different specificities to counteract the diverse forms of PGs existing in nature. Regardless of origin, PGIPs can exhibit different inhibitory activities to the same or different pathogens (Liu et al. 2017). A single PGIP may display different mechanisms of PG inhibition (competitive, non-competitive and mixed) suggesting that the protein is highly versatile in recognizing different epitopes of PGs (Barmore and Nguyen 1985; King et al. 2002; Sicilia et al. 2005). Besides a single or a few amino acid residues, the inhibition specialization of PGIPs were mainly concerned with its binding sites with PGs (Benedetti et al. 2013; Leckie et al. 1999). The low resolution structure of the PvPGIP2-FvPG complex shows that PvPGIP2 contacts the enzyme using the concave surface of its LRR solenoid in a head-to-head orientation. Both the N- and C-terminal perimeters of the active site cleft of FvPG (produced by *Fusarium verticillioides*) engaged at the PvPGIP2-FvPG complex, whereas CluPG1 from *Colletotrichum lupini* was engaged only at the C-terminal border (Benedetti et al. 2013). In addition, the loops surrounding the active site cleft of FvPG also performed the function in contact with PvPGIP2 (Benedetti et al. 2013). In our study, we found that OsPGIP2 bound to the loop at the C-terminal edge of RsPG1, but not the active site in the cleft (see Fig. 2c). This means that the inhibitory mechanism of OsPGIP2 for RsPG1 is non-competitive, which is also the case for OsPGIP1 (see Fig. 2b).

Site-directed mutagenesis has shown that the residues involved in the interaction between PGIPs and PGs are located in the concave surface of PGIPs (Spinelli et al. 2009; Benedetti et al. 2011a, 2011b, 2013). A single amino acid substitution allowed PGIPs to acquire or lose recognition for PGs. When eight amino acids of PvPGIP2 were replaced with the corresponding amino acids of PvPGIP1, each mutation caused a decrease in the affinity for PGs in *Fusarium moniliforme* and *Aspergillus niger* (Leckie et al. 1999). Among these amino acids, residue Q253 made a major contribution and its replacement with a lysine led to a dramatic reduction in the binding energy of the PvPGIP2-FmPG complex. Conversely, when amino acid K253 was mutated to a glutamine, which is present in PvPGIP2, PvPGIP1 acquired the ability to interact with FmPG (Leckie et al. 1999). In OsPGIP2, the 233rd amino acid may be involved in PGs inhibition. When the leucine located in the seventh LRR was substituted with phenylalanine, OsPGIP2 acquired inhibitory activity for RsPGs and reduced enzyme activity by over 40% (see Fig. 5). However, when the L233 was substituted with phenylalanine, P17 in signal peptide area and N327 in the C-terminal were substituted with proline and serine respectively at the same time. Whether the substitution of non-target amino acids in the mutant would enhance or weaken its inhibitory effect on RsPGs remains to be further verified**.** Certainly, many researchers thought that the amino acids of PGIPs involved in the interaction of PGIPs-PGs were located in the concave surface, which was consist of the *β-*sheet B1, but not in other domains (Leckie et al. 1999; Federici et al. 2001; Benedetti et al. 2011a, 2011b; Kalunke et al. 2015). This statement was supported further by the desolvation energy calculations and alanine scanning assay (Casasoli M et al., 2009).

Many *PGIP* genes have been transferred into other crop species and overexpression has enhanced disease resistance (Ferrari et al. 2012; Bashi et al. 2013; Kalunke et al. 2015; Wang et al. 2015; Chen et al. 2016). Previous studies reported that overexpression of *OsPGIP1* and *OsPGIP4* in rice resulted in an increase in disease resistance for SB and bacterial leaf streak, respectively (Wang et al. 2015; Chen et al. 2016; Feng et al. 2016). In this study, we found that OsPGIP2 had no inhibitory for RsPGs unless L233 was substituted with phenylalanine. Overexpression of *OsPGIP2*^*L233F*^ in the commercially susceptible japonica cv. XD 3 enhanced resistance to sheath blight in the field and reduced the tissue degradation capability of RsPGs (Fig. 5). Furthermore, compared to wild-type plants, the overexpression *OsPGIP2*^*L233F*^ had no impact on morphological and developmental traits associated with yield. In conclusion, OsPGIP2^L233F^ has important practical implications for improving SB resistance in rice.

## Materials and methods

### Plant materials and growth conditions

Seeds of cv. XD3 and its transgenic derivatives were surface-sterilized before germination by incubating in sodium hypochlorite (0.5% v/v) for 10 min, then rinsed thoroughly in sterile water. Rice seedlings were grown in the greenhouse with a 14 h photoperiod at 25 °C, and seedlings at the five-leaf stage were selected for DNA and RNA extraction. The plants used for inoculation were sown in the field with normal nutrient and water management.

### Nucleic acid extraction and gene cloning

Genomic DNA was extracted using the Plant DNA Isolation Kit (Sigma). Total RNA was extracted using the RNAiso Plus reagent according to the manufacturer’s instruction (Takara). First strand cDNA synthesis was conducted using PrimeScript II reverse transcriptase as recommended (Takara). Full-length *OsPGIP2* was obtained by PCR with the primers *OsPGIP2*-F (5′-ATACACGGCATTGCATGCAC-3′) and *OsPGIP2*-R (5′-CTTACACTCGTTCTCCGTAC-3′) and both genomic DNA and cDNA were used as templates. The amplification conditions were as follow: 5 min at 94 °C; 30 cycles at 94 °C for 30 s, 62 °C for 30 s, and 72 °C for 1 min; and a final step at 72 °C for 7 min. PCR products were amplified and ligated into pMD19-T for sequencing. Three clones of *OsPGIP2* were independently sequenced using an automated DNA sequencer (ABI PRISM™ 3730XL DNA Analyzer). Sequence reads were assembled by BioEdit 5.0.9 (Hall 1999).

### Sequence analysis

Bioinformatic analyses of open reading frames (ORFs), introns, exons, deduced amino acid sequences, molecular weights, and pIs were performed using the Vector NTI Suite 8.0 software package. Gene ontology was predicted using PredictProtein (https://www.predictprotein.org/). Identification of signal peptide cleavage sites was performed using the online tool (http://www.cbs.dtu.dk/services/SignalP/) (Bendtsen et al. 2004). The solubility, hydropathicity, instability, estimated half-life and the aliphatic index of the recombinant protein were predicted using tools Recombinant Protein Solubility Prediction (http://biotech.ou.edu/) (Diaz et al. 2009) and ExPASy ProtParam tool (http://au.expasy.org/tools/protparam.html) (Gasteiger et al. 2005). Transmembrane helices and subcellular localization analysis were performed using DTU Bioinformatics Prediction Servers (http://www.cbs.dtu.dk/services/). Secondary and tertiary structures of deduced proteins were predicted using HNN SECONDARY STRUCTURE PREDICTION METHOD (http://npsa-pbil.ibcp.fr/cgi-bin/npsa_automat.pl? page=/NPSA/npsa_hnn.html) (Combet et al. 2000) and SWISS-MODEL (http://swissmodel.expasy.org/) (Arnold et al. 2006), respectively. Protein-protein docking was predicted using Cluspro 2.0 (https://cluspro.bu.edu/).

### Site directed mutagenesis

Primers OsP2-F (5′-ATACACGGCATTGCATGCAC-3′) and OsP2-R (5′-CTTACACTCGTTCTCCGTAC-3′) were used to amplify full-length *OsPGIP2*. OsP2_L233F_-F (5′-TGCTGGCGGCGGCGAACCTGGCGTTCGT-3′)/OsP2_L233F_-R (5′- GAACGCCAGGTTCGCCGCCGCCAGCAGGTCCGC-3′), OsP2_R253Q_-F (5′-CGGCGGTGTTCCGGCGGGCACAATAC-3′)/OsP2_R253Q_-R (5′- ACAGGTCCACGTATTGTGCCCGCCG-3′) and OsP2_Q300H_-F (5′-TGCATACGTTCAACGTCAGCTACAACAAGA-3′)/OsP2_Q300H_-R (5′-TGTTGTAGCTGACGTTGAACGTATGCAG-3′) were designed to introduce mutations in OsPGIP2 at the 233th (L233F), 253th (R253Q) and 300th (Q300H) amino acid residues, respectively. The mutated genes were sequenced to confirm the desired mutations.

### Overproduction of OsPGIP2 and mutant derivatives in *Escherichia coli*

For overexpression in *E. coli*, full-length *OsPGIP2* encoding the mature protein and mutant forms were amplified using primers OsP2-PE-F (5′-ttcCATATGTGACCATGGATGTGAAGCTCC-3′, *Nde*I site is underscored)/OsP2-PE-R (5′-ccgCTCGAGAGTATTATTTATCGACGACGGCA-3′, *Xho*I site is underscored). Products were digested using *Nde*I and *Xho*I and subcloned into the protein expression vector pET-22b (+) to generate His-tagged fusion protein expression construct; these constructs were then introduced into *E. coli* strain BL21 for overexpression as fusion proteins after induction with isopropyl β-D-1-thiogalactopyranoside. *E. coli* BL21 cells were harvested by centrifugation at 10,000 rpm for 10 min at 4 °C and then disrupted by sonication at 200 W (Chen et al. 2016). Refolding of recombinant OsPGIP2 and mutant forms present in protein inclusion bodies was performed as described by De Bernardez (1998). Inclusion body proteins were acquired and purified by centrifuging at 12,000 rpm for 10 min at 4 °C, washing with 1% TritonX-100, and then solubilized at Tris buffer containing 8 M urea (pH 8.0). After being agitated intensely for 2–3 h, the mixture was centrifuged at 12,000 rpm for 10 min at 4 °C, and phosphate buffer solution was added into the supernatant and the mixture was loaded dialysis bag to dialyze for 20 h at 4 °C. Recombinant His-tagged fusion proteins were purified and collected according the manufacturer’s instructions for His-Bind Resin (Novagen); proteins were then lyophilized and stored at − 70 °C until needed.

### Inhibiting activities of OsPGIP2 and its mutations on RsPGs

The extraction and purification of RsPGs from *R. solani* was performed as described previously (Chen et al. 2010). The inhibition of RsPG activity by OsPGIP2 and mutant proteins was evaluated using the reduced sugar assay and diffusion assays (Chen et al. 2016**;** Taylor and Secor 1988). In the reduced sugar assay, 2.0 mL of 0.1 g OsPGIP2 or mutated protein was added to mixtures containing 0.5 mL of RsPGs at 0.2 g/mL, 0.5 mL of 0.25% polygalacturonic acid (Sigma) in 50 mM sodium citrate buffer at pH 5.0, and 1 mL redistilled water. This solution was incubated at 37 °C for 1 h, 2.5 mL of dinitrosalicylate was added, and the mixture was incubated in boiling water for 10 min. After cooling, the optical density values of solutions were measured by UV spectrophotometry; one enzyme unit was defined as the amount of enzyme required to release 1 μg of reduced sugar at 37 °C in 1 h.

The diffusion assay was also used to evaluate inhibition of RsPG activity by OsPGIP2 and mutant proteins. Wells (5 mm diameter) were excised in medium containing 0.8% type II agarose and 0.5% polygalacturonic acid in 0.1 M NaAC buffer (pH 4.7); RsPGs and OsPGIP2 or mutant proteins were then added to the well. After incubation at 37 °C for 17 h, the medium was stained with ruthenium red (0.05% w/v in water) for 2 h and then rinsed thoroughly with water. The inhibitory activities of OsPGIP2 and mutant proteins were evaluated based on the diameter of the diffusion rings.

### Expression of *OsPGIP2*^*L233F*^ in transgenic rice

Full-length *OsPGIP2*^*L233F*^ was amplified using primer pairs OsP2-OE-F (5′-ggGGTACCTGACCATGGATGTGAAGCTCC-3′, *Kpn*I site is underscored)/OsP2-OE-R (5′-cGAGCTCAGTATTATTTATCGACGACGGCA-3′, *Sac*I site is underscored) and cloned into the binary vector pCAMBIA 1301. Expression of *OsPGIP2*^*L233F*^ was driven by the maize ubiquitin-1 promoter and terminated by the NOS termination sequence in construct pCAMBIA 1301-*OsPGIP2*^*L233F*^. The overexpression construct was introduced into *Agrobacterium tumefaciens* EHA105 using the freeze-thaw method (Sambrook and Russell 2001) and introduced into rice cv. XD3 via *Agrobacterium*-mediated transformation (Chern et al. 2005). Transgenic plants were confirmed by PCR using primers HygRT-F (5′-ATTTGTGTACGCCCACAGT-3′) and HygRT-R (5′-GGATATGTCCTGCGGGTAAA-3′), which are specific to the hygromycin phosphotransferase gene (*HPT*). The quantitative reverse-transcription-PCR primers used in this paper were Osp2-RT-F (5′-GGTCGTCGTTCTTGTGCTCG-3′) and Osp2-RT-R (5′-GGTGGTGTCGTCGCAGGTGA-3′), which resulted in a fragment of 190 bp. The PCR primers specific to *OsACTIN1*, used as the internal control gene, were according to the report by Chen et al. (2016). Each reaction was repeated three times. Two biological replicates for each sample were included.

### Evaluation of SB resistance and agronomic traits in transgenic lines

Twenty T2 homozygous plants originating from each T1 parental line were planted in the field and screened at the seedling stage. DNA extracted from leaf sections was used as a template to detect *OsPGIP2*^*L233F*^ with primers HygRT-F/HygRT-R. Homozygous transgenic lines and wild-type cv. XD3 were planted in the field in a completely randomized design with three replications for evaluation of agronomic traits including heading date, plant height, flag leaf length and width, tiller number, number of productive panicles, panicle length, number of primary branches, seed numbers/panicle, grain length, grain width, and 1,000-grain weight (1,000 GW). All traits were measured as described by Zuo et al. (2007).

To test whether *OsPGIP2*^*L233F*^ expression increased resistance to tissue degradation caused by the RsPGs, a tissue necrosis assay was performed as described previously (Chen et al. 2017). Sheaths of rice seedlings were cut into 2 mm^2^ segments, immersed in the solution containing RsPGs, and then evaluated for reducing sugar content and electrical conductivity. The virulent *R. solani* YN-7 was used to inoculate transgenic homozygous lines, and disease severity was evaluated by averaging the disease severity scores on 30 or more tillers (Zuo et al. 2013**;** Chen et al. 2016).

### Statistical analyses

Data were analyzed using the one-way analysis of variance procedure in the SPSS 12.05 program. Mean differences were compared by the Tukey’s test, and *P* values < 0.05 were considered significant.

## Conclusion

In summary, we identified the important amino acid residues of OsPGIP2, and the L233F plays an important role for OsPGIP2 improving SB resistance in rice.

## Additional files

Additional file 1: **Figure S1.** Screening of 30 putative transgenic rice lines transformed with pCAMBIA 1301-OsPGIP2^L233F^. Lines were screened by PCR for the presence of the *HPT* gene encoding hygromycin resistance. Abbreviations: M, molecular marker; P, positive control, pCAMBIA 1301; N, negative control, ddH_2_O; Tr01-Tr30, different transgenic lines. (PDF 195 kb)

Additional file 2: **Table S1.** Segregation ratio of the markers (*HPT gene*) in the transgenic population of overexpressing *OsPGIP2*^*L233F*^. (DOCX 44 kb)

## Data Availability

The datasets supporting the conclusions of this article are included within the article and its additional files.

## Abbreviations

kD: Kilodalton
PGIPs: Polygalacturonase inhibiting proteins
PGs: Polygalacturonases
QTL: Quantitative trait loci
SB: Sheath blight
