# Supplementary figures and images for: Amino acid substitutions in a polygalacturonase inhibiting protein (OsPGIP2) increases sheath blight resistance in rice
Source: Rice (N Y). 2019 Jul 29;12:56. doi: 10.1186/s12284-019-0318-6 (PMC6663954; doi:10.1186/s12284-019-0318-6)

Supplemental Fig. 1

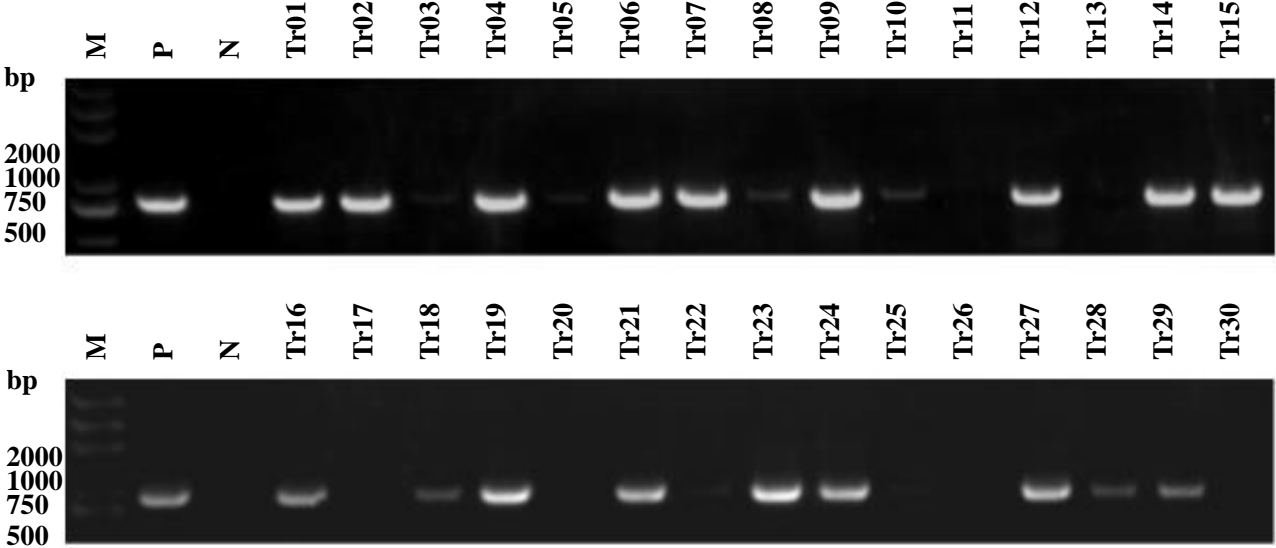

Supplement: Supplementary file 1 — Figure S1. Screening of 30 putative transgenic rice lines transformed with pCAMBIA 1301-OsPGIP2L233F. Lines were screened by PCR for the presence of the HPT gene encoding hygromycin resistance. Abbreviations: M, molecular marker; P, positive control, pCAMBIA 1301; N, negative control, ddH2O; Tr01-Tr30, different transgenic lines. (PDF 195 kb) [file 12284_2019_318_MOESM1_ESM.pdf]
