# Supplementary material for: Amino acid substitutions in a polygalacturonase inhibiting protein (OsPGIP2) increases sheath blight resistance in rice
Source: Rice (N Y). 2019 Jul 29;12:56. doi: 10.1186/s12284-019-0318-6 (PMC6663954; doi:10.1186/s12284-019-0318-6)
Supplement: Supplementary file 2 — Table S1. Segregation ratio of the markers (HPT gene) in the transgenic population of overexpressing OsPGIP2L233F. (DOCX 44 kb) [file 12284_2019_318_MOESM2_ESM.docx]

**Table S1.** Segregation ratio of the markers (*HPT gene*) in the transgenic population of overexpressing *OsPGIP2^L233F^*

| Transgene line* | Number of tested plants | Number of positive plants | Number of negative plants | χ^2^ | P-value |
| --- | --- | --- | --- | --- | --- |
| Trans 01 | 67 | 39 | 28 | 5.2211 | 0.0222 |
| Trans 02 | 70 | 41 | 29 | 5.1274 | 0.0236 |
| **Trans 03** | **72** | **55** | **17** | **0.0438** | **0.8339** |
| Trans 04 | 69 | 43 | 26 | 3.1160 | 0.0775 |
| Trans 05 | 71 | 47 | 24 | 1.5738 | 0.2096 |
| Trans 06 | 72 | 47 | 25 | 1.9189 | 0.1660 |
| Trans 07 | 72 | 41 | 31 | 6.2149 | 0.0127 |
| **Trans 08** | **72** | **54** | **18** | **0.0000** | **1.0000** |
| Trans 09 | 71 | 59 | 12 | 1.6061 | 0.2051 |
| **Trans 10** | **69** | **51** | **18** | **0.0254** | **0.8744** |
| Trans 11 | 69 | 39 | 30 | 6.3502 | 0.0117 |
| Trans 12 | 70 | 45 | 25 | 2.2768 | 0.1313 |
| Trans 13 | 72 | 49 | 23 | 1.0034 | 0.3166 |
| Trans 14 | 71 | 47 | 24 | 1.5738 | 0.2096 |
| Trans 15 | 71 | 43 | 28 | 4.0460 | 0.0443 |
| Trans 16 | 68 | 48 | 20 | 0.4017 | 0.5261 |
| **Trans 17** | **70** | **52** | **18** | **0.0111** | **0.9165** |
| Trans 18 | 68 | 48 | 20 | 0.4017 | 0.5261 |
| Trans 19 | 72 | 50 | 22 | 0.6506 | 0.4198 |
| Trans 20 | 69 | 40 | 29 | 5.4453 | 0.0196 |
| Trans 21 | 71 | 27 | 44 | 23.5802 | 0.0000 |
| **Trans 22** | **70** | **53** | **17** | **0.0113** | **0.9165** |
| Trans 23 | 71 | 49 | 22 | 0.7464 | 0.3878 |
| Trans 24 | 72 | 39 | 33 | 8.1292 | 0.0044 |
| Trans 25 | 70 | 45 | 25 | 2.2768 | 0.1313 |
| Trans 26 | 67 | 37 | 30 | 7.1038 | 0.0077 |
| Trans 27 | 71 | 43 | 28 | 4.0460 | 0.0443 |
| Trans 28 | 69 | 39 | 30 | 6.3502 | 0.0117 |
| **Trans 29** | **67** | **50** | **17** | **0.0030** | **0.9563** |
| Trans 30 | 71 | 40 | 31 | 6.5657 | 0.0104 |

*Rows marked in bold face font indicate lines segregating at a 3:1 ratio of *HPT* gene in the T1 population.
